# System x_c_^−^ in microglia is a novel therapeutic target for post-septic neurological and psychiatric illness

**DOI:** 10.1038/s41598-019-44006-8

**Published:** 2019-05-17

**Authors:** Yoshinori Kitagawa, Kazuhiro Nakaso, Yosuke Horikoshi, Masaki Morimoto, Takuma Omotani, Akihiro Otsuki, Yoshimi Inagaki, Hideyo Sato, Tatsuya Matsura

**Affiliations:** 1 Division of Medical Biochemistry, Department of Pathophysiological and Therapeutic Science, Tottori University Faculty of Medicine, Yonago, 683-8503 Japan; 2 Division of Anesthesiology and Critical Care Medicine, Department of Surgery, Tottori University Faculty of Medicine, Yonago, 683-8503 Japan; 3 Division of Surgical Oncology, Department of Surgery, Tottori University Faculty of Medicine, Yonago, 683-8503 Japan; 4 Laboratory of Biochemistry and Molecular Biology, Department of Medical Technology, Niigata University, Niigata, 950-2181 Japan

**Keywords:** Depression, Therapeutics, Dementia

## Abstract

Post-septic neurological and psychiatric illness (PSNPI) including dementia and depression may be observed after sepsis. However, the etiology of PSNPI and therapeutic treatment of PSNPI are unclear. We show that glutamate produced from microglia through the activity of system x_c_^−^ plays a role in PSNPI. We established a mouse model of PSNPI by lipopolysaccharide (LPS) treatment that shows a disturbance of short/working memory and depression-like hypoactivity. Glutamate receptor antagonists (MK801 and DNQX) reduced these phenotypes, and isolated microglia from LPS-treated mice released abundant glutamate. We identified system x_c_^−^ as a source of the extracellular glutamate. xCT, a component of system x_c_^−^, was induced and expressed in microglia after LPS treatment. In xCT knockout mice, PSNPI were decreased compared to those in wildtype mice. Moreover, TNF-α and IL-1β expression in wildtype mice was increased after LPS treatment, but inhibited in xCT knockout mice. Thus, system x_c_^−^ in microglia may be a therapeutic target for PSNPI. The administration of sulfasalazine, an inhibitor of xCT, in symptomatic and post-symptomatic mice improved PSNPI. Our results suggest that glutamate released from microglia through system x_c_^−^ plays a critical role in the manifestations of PSNPI and that system x_c_^−^ may be a therapeutic target for PSNPI.

## Introduction

Post-septic neurological and psychiatric illnesses, such as dementia, hypokinesia, depression, and delirium, are critical complications that can affect functional prognosis^1,2^. Sepsis is a complex syndrome characterized by an enhanced innate immune response to bacterial infection. Lipopolysaccharide (LPS) endotoxin within the bacterial wall triggers a release of proinflammatory mediators from macrophages and neutrophils through a Toll-like receptor 4 signaling pathway that mediates host damage^3^. Although a low dose of LPS does not cross the blood-brain barrier (BBB)^4,5^, activation of microglia in the central nervous system (CNS) is observed in peripherally LPS-treated models^6^. Furthermore, CNS-related symptoms such as depression-like hypoactivity and memory disorder may be observed after sepsis even though obvious neuronal death is not detected^7^. In cases with neuronal death or brain tissue necrosis, such as that induced by high dose LPS administration, damage-associated molecular patterns (DAMPs) may play a critical role in the pathogenesis^8,9^. However, in cases without obvious neuronal death, such as mild endotoxin shock induced by low dose LPS, the etiology of neurological and/or psychiatric illness in the post-septic stage has not been explained clearly.

The role of microglia in the pathogenesis of neurological and psychiatric disorders has been examined in recent years^10,11^. Activated microglia are observed not only in the brain of individuals with inflammatory disorder, but also in the brain of individuals with neurodegenerative and/or psychiatric diseases^10,11^. Activated microglia take on an amoeboid shape with phagocytic activity and increase their gene expression of neurotoxic mediators, such as proinflammatory cytokines, nitric oxide, and proteases^12^. Recent reports have demonstrated that excitatory amino acid glutamate is released from microglia in the brain of individuals with neurological diseases^13,14^.

A classic hypothesis of glutamate’s association with depression and memory disorder is the “Theory of Glutamate^15,16^”. Extracellular glutamate is excitotoxic for neuronal cells, and glutamate receptors, such as *N*-methyl-D-aspartate (NMDA) receptor and α-amino-3-hydroxy-5-methyl-4-isoxazolepropionic acid (AMPA) receptor, are expressed abundantly in the brain^17,18^. Although the primary origin of released glutamate in the pathogenesis of neurological and psychiatric diseases is thought to be from neuronal cells, other type of cells such as glial cells can also release glutamate. Therefore, the all sources of extracellular glutamate in the brain have not been established.

System x_c_^−^ is transmembrane sodium-independent and chloride-dependent antiporter of cystine and glutamate. It consists of the 4F2 heavy chain (4F2hc; CD98; Slc3a2) and xCT (Slc7a11), a specific molecule for system x_c_^−^^19–21^. xCT is expressed constitutively in immune-related organs, such as spleen, thymus, and bone marrow. However, in the CNS, the expression level of xCT is quite low in normal conditions^22^. xCT is an inducible molecule that is dependent on the transcriptional activation of NF-E2-related factor-2 (Nrf2) or hypoxia inducible factor-1α (Hif-1α)^23,24^. For instance, under oxidative stress, increased xCT can play an important role in the synthesis of the antioxidant molecule glutathione (GSH). System x_c_^−^ transports extracellular cystine into the cells under the condition of oxidative stress and is the cysteine, prepared by the reduction of cysteine, is required for the major component of antioxidant GSH^19–21^. Although system x_c_^−^ provides the resistance against oxidative stress^25^, it may affect adversely the surrounding neurons because of glutamate released in the CNS^26,27^.

In order to clarify the occurrence of post-septic neurological and psychiatric illness (PSNPI), we investigated the role and origin of glutamate in neurological and psychiatric symptoms using mice treated with a systemic administration of low dose LPS. Here, we report the importance of system x_c_^−^ in microglia as a source of glutamate in the pathogenesis of post-septic memory disorder and depression-like hypoactivity. Furthermore, we also examined the therapeutic effect of xCT inhibitor and evaluated the inhibition of system x_c_^−^ as a novel therapeutic target of these neurological and psychiatric symptoms.

## Results

### Systemic administration of LPS activates microglia and causes depression-like hypoactivity and memory disorder

**Figure 1:**
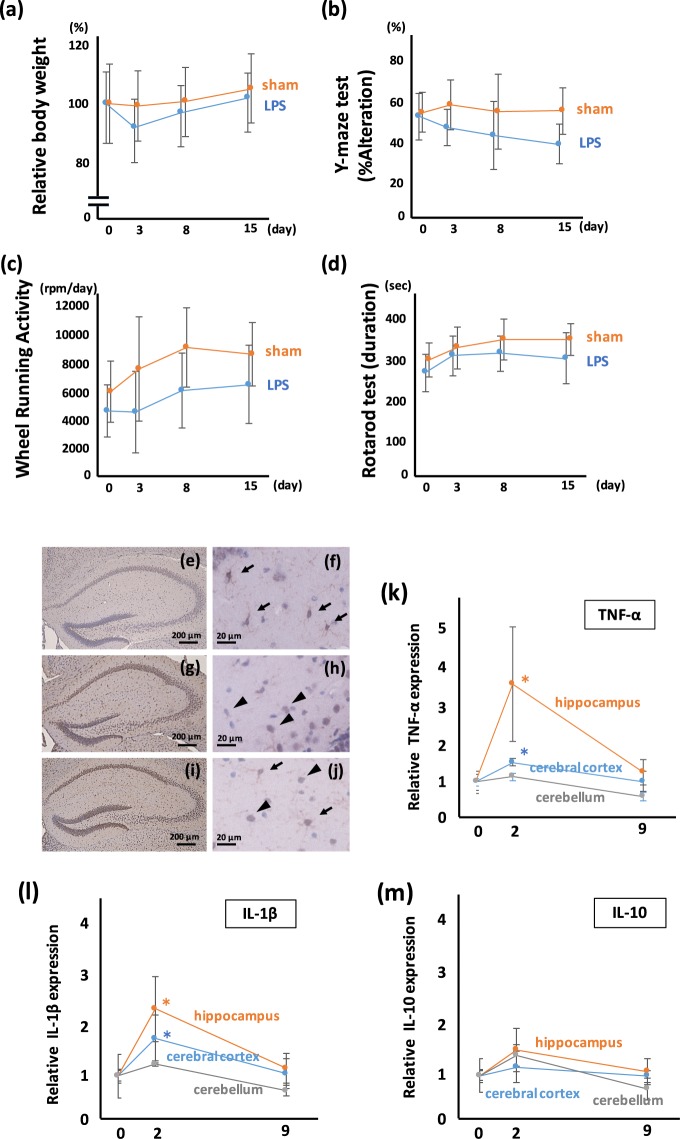
Systemic treatment with low dose LPS induces neurological and psychiatric illness with microglial activation. (**a**) Change of BW after systemic LPS administration. The transient loss, but not statistically significant, of BW in LPS-treated mice at day 3 after LPS administration is observed. BW of LPS-treated mice are recovered gradually. F (1, 110) = 0.754, p = 0.39. (**b**) Y-maze test for measuring short memory and working memory. The Y-maze score of LPS-treated mice is significantly poorer than that of sham-treated mice. The reduction of the Y-maze score continued during the experiment. F (1, 110) = 22.17, p < 0.0001. (**c**) WRA for evaluating activity and anxiety. The WRA score of LPS-treated mice is significantly reduced, during the experiment. F (1, 110) = 20.61, p < 0.0001. (**d**) RR test for evaluating motor coordination. The RR score of LPS-treated mice is significantly reduced after LPS treatment. F (1, 110) = 11.21, p < 0.001. (**a–d**; sham; n = 13, LPS; n = 15) (**e–j**) Microglial staining using Iba-1 antibody in the hippocampus. (**e**,**f**) In sham-treated mice, the density of Iba-1 signal is not high, and ramified-shaped microglia are observed at high magnification (arrows). (**g,h**) In the hippocampus from mice 2 days after LPS treatment, the Iba-1 signal is dense, and amoeboid-shaped microglia are observed at high magnification (arrowheads). (**i**,**j**) In the hippocampus from mice 15 days after LPS treatment, the Iba-1 signal is dense, and both amoeboid-shaped (arrowheads) and ramified-shaped microglia (arrows) are observed at high magnification. Time course analyses for (**k**) TNF-α, (**l**) IL-1β, and (**m**) IL-10. *p < 0.01 vs day 0 mice (sham-treated mice). TNF-α and IL-1β expression in the hippocampus and cerebral cortex are increased at 2 days after LPS treatment, followed by a gradual decrease. TNF-α and IL-1β expression in the cerebellum are mildly increased, but the increase is not statistically significant. IL-10 is mildly increased in the hippocampus, cerebral cortex, and cerebellum at 2 days after LPS treatment, but the increase is not statistically significant. (**k–m**; sham-treated; n = 7, LPS-treated 2 days; n = 7, LPS-treated 9 days; n = 3).

First, we established the mouse model with LPS-induced depression and memory disturbance. Four types of behavioral tests were used: (i) the change of body weight (BW) as a parameter of appetite; (ii) the Y-maze spontaneous alteration test (Y-maze) as a parameter of recent memory and working memory; (iii) the wheel running activity (WRA) as a parameter of hyper/hypoactivity and anxiety; and (iv) the rotarod test (RR) as a parameter of coordination and skill learning. BW was reduced transiently at the point of day 3 in LPS-administered mice, however, the difference of BW between sham and LPS-administered mice disappeared gradually (Fig. 1a). The result of the Y-maze showed a significant disturbance in recent memory and working memory in the LPS-administered mice (Fig. 1b). The WRA showed hypoactivity and mice treated with LPS showed a tendency to remain in the dark box, suggesting depression-like hypoactivity (Fig. 1c). The RR score of LPS-treated mice is significantly reduced at 15 days after LPS treatment (Fig. 1d). Together, these results demonstrate that the mice administered LPS show depression-like hypoactivity and memory disturbance.

Histological analyses by hematoxylin-eosin (HE) staining did not show obvious neuronal death in the hippocampus, cerebral cortex, and cerebellum (Supplementary Fig. S1). We also confirmed the activation of microglia in the brain after the administration of low dose LPS. Iba-1 staining, that indicates the activation of microglia, was enhanced after LPS treatment (Fig. 1e,g,i). As reported previously, the shape of microglia is observed as ramified-type in resting state (Fig. 1f), and an amoeboid-type in mice administered with LPS (Fig. 1h,j). In the hippocampus from the mouse 15 days after LPS treatment, both ramified-type and amoeboid-type microglia were observed (Fig. 1j).

The proinflammatory cytokines TNF-α (Fig. 1k) increased in the hippocampus and cerebral cortex at 2 days after LPS administration and decreased gradually to the base line at day 9. Similarly, IL-1β (Fig. 1l) increased in the hippocampus and cerebral cortex, and cerebellum at 2 days after LPS administration and decreased gradually to the base line, while the anti-inflammatory cytokine IL-10 (Fig. 1m) did not change significantly in our experimental conditions.

### Extracellular glutamate is associated with the behavioral disturbance of mice treated with LPS

**Figure 2:**
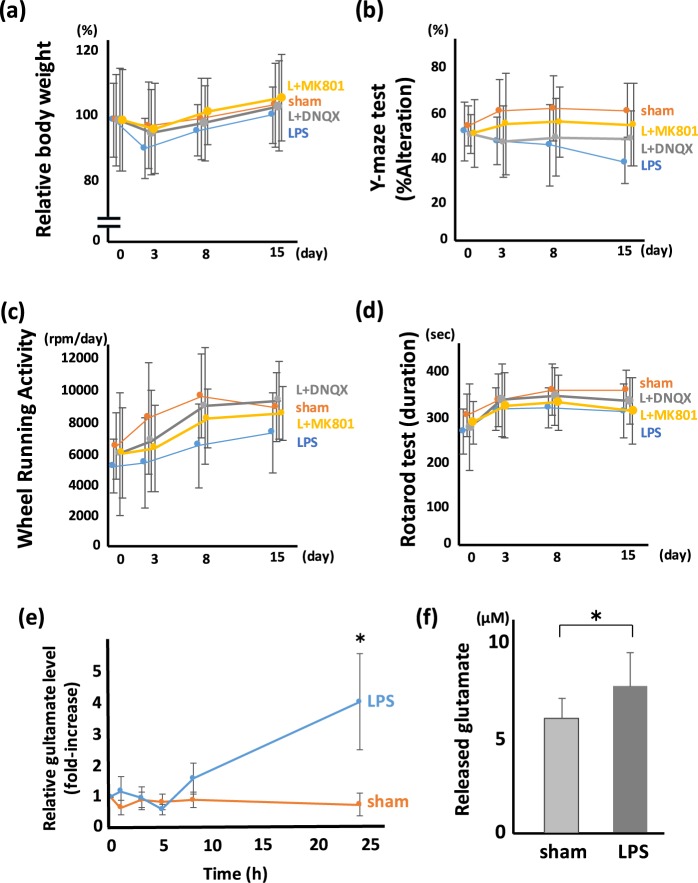
Glutamate released from microglia is a trigger for post-septic neuronal and psychiatric illness. The effect of glutamate receptor antagonist on PSNPI. Behavioral test including (**a**) BW, (**b**) Y-maze, (**c**) WRA, and (**d**) RR with/without glutamate receptor antagonist. (**a–d**) Sham; n = 12, LPS; n = 13, LPS + DNQX (L + DNQX); n = 9, LPS + MK801 (L + MK801); n = 11. (**a**) MK801 and DNQX show a tendency to improve BW loss at 3 days after LPS treatment, but the differences are not statistically significant. F (3, 176) = 0.68, p = 0.56. (**b**) MK801 prevents the reduction of the Y-maze score significantly. F (3, 176) = 6.38, p < 0.001. Post hoc; LPS vs L + MK801, p < 0.01. (**c**) DNQX and MK801 prevents the reduction of WRA score significantly. F (3, 176) = 5.39, p < 0.005. Post hoc; LPS vs L + DNQX, p < 0.01; LPS vs L + MK801, p < 0.05. (**d**) The effect of MK801 and DNQX on the RR score is mild and not statistically significant. F (3, 176) = 2.85, p = 0.039. Post hoc; LPS vs L + DNQX, p = 0.15; LPS vs L + MK801, p = 0.45. (**e**) Relative glutamate level in extracellular space (*in vivo* microdialysis). Extracellular glutamate gradually increased from 8 h after LPS treatment, and statistically high level of glutamate was shown at 24 h after LPS treatment. (*p < 0.01, sham; n = 4, LPS; n = 4) (**f**) Released glutamate from microglia. Isolated microglia were incubated with cysteine/cystine in HBSS. Microglia isolated from LPS-treated mice release more glutamate than those from sham-treated mice. (*p < 0.01, sham; n = 14, LPS; n = 15).

Glutamate has been reported to play an important role in neurological/psychiatric behavioral abnormalities. Thus, we investigated the association of glutamate with behavioral disturbance induced by LPS administration (Fig. 2a–d). We used an antagonist for NMDA and AMPA receptors, MK801 and 6,7-dinitroquinoxaline-2,3-dione (DNQX), respectively, to examine the association between glutamate and depression-like hypoactivity and memory disturbance. The administration of MK801 and DNQX showed a tendency to improve the loss of BW, but it was not statistically significant (Fig. 2a). In the Y-maze, MK801 significantly inhibited the memory disturbance induced by LPS (Fig. 2b), while DNQX tended to inhibit the memory disturbance, but the change was not significant. In WRA, DNQX improved the hypoactivity induced by LPS significantly, while MK801 tended to inhibit the hypoactivity, but the change was not significant (Fig. 2c). In RR, no significant difference was observed with MK801 or DNQX administration (Fig. 2d).

In order to confirm whether glutamate was actually released to extracellular space after LPS administration, we measured extracellular glutamate using *in vivo* microdialysis system. Although the increase of extracellular glutamate was not observed until 5 h after LPS treatment, mild increase of extracellular glutamate observed at 8 h after LPS administration, and glutamate level was reached 4-fold at 24 h after LPS treatment (Fig. 2e). Furthermore, we quantitated the release of glutamate from isolated microglia. The level of glutamate released by microglia isolated from LPS-treated mice was significantly higher than that released by microglia isolated from sham-treated mice (Fig. 2f). It was similar result even in the examination which divided the cerebral cortex and hippocampus (Supplementary Fig. S2).

### System x_c_^−^ expressed in microglia is an important source of extracellular glutamate

**Figure 3:**
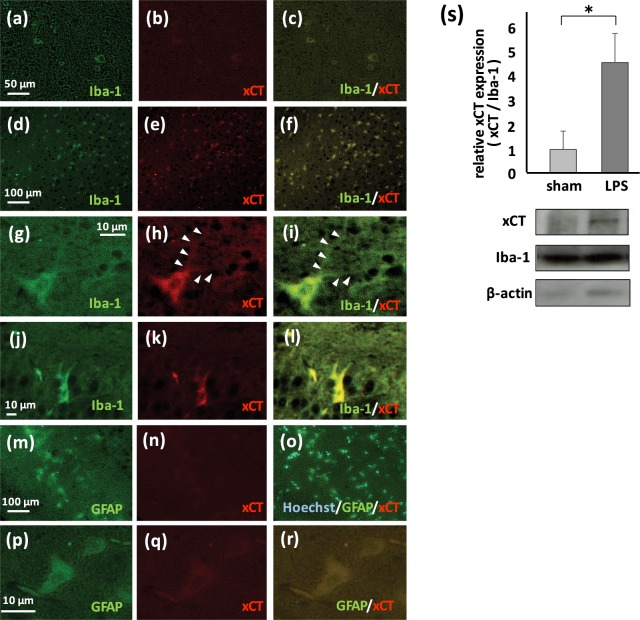
xCT, a special component of system x_c_^−^, is induced in microglia by LPS. (**a,d,g,j**) Immunofluorescent staining using Iba-1 antibody. (**b,e,h,k,n,q**) Immunofluorescent staining using xCT antibody. (**m,p**) Immunofluorescent staining using GFAP antibody. (**c,f,i,l,o,r**) Merged images. (**a–c**) Low magnification image of cerebrum sampled from sham-treated mouse at 2 days after administration. Iba-1 and xCT are co-localized, however, expression level of xCT is slight. (**d–f**) Low magnification image of cerebrum sampled from LPS-treated mouse at 2 days after administration. Iba-1 and xCT are co-localized. (***g–i***) High magnification image of cerebrum. xCT is expressed in Iba-1-positive ramified microglia with protuberances (arrowheads). (**j–l**) High magnification image around dentate gyrus in the hippocampus. xCT is expressed in Iba-1-positive amoeboid microglia*. (***m–o***)* Low magnification image of cerebral cortex sampled from LPS-treated mouse at 2 days after administration. GFAP and xCT are co-localized weakly. (**p–r**) High magnification image of the brain stem. xCT is weakly expressed in GFAP-positive reactive astrocytes at 15 days after LPS administration. (**s**) Induction of xCT in microglia derived from LPS-treated mice (*p < 0.01, sham; n = 6, LPS; n = 6). Representative immunoblot for xCT, Iba-1, and β-actin are also shown.

Previous reports have suggested that system x_c_^−^ and gap junction hemichannel play roles in the release of glutamate^27,28^. We investigated the expression of xCT in the brain using immunofluorescent staining. In sham-treated mouse brain, quite low level of xCT was observed in cells expressing Iba-1, a major marker of microglia (Fig. 3a–c). Immunohistochemistry indicated that inducible expression of xCT was observed mainly in Iba-1positive microglia (Fig. 3d–l), while weak induction of xCT was observed in GFAP-positive astrocytes (Fig. 3m–r). This induction of xCT in astrocytes was observed in later phase (day 15, Fig. 3p–r), The expression of xCT in neurons and oligodendrocytes was not detected.

We measured inducible expression of xCT in microglia isolated from sham- and LPS-treated mice using western blotting. Although the expression of xCT in the resting state (sham-treatment) was quite low, a 4-fold increase in the expression of xCT was detected in microglia from LPS-treated mice (Fig. 3s).

### Deficiency of xCT reduces LPS-induced behavioral disturbance

**Figure 4:**
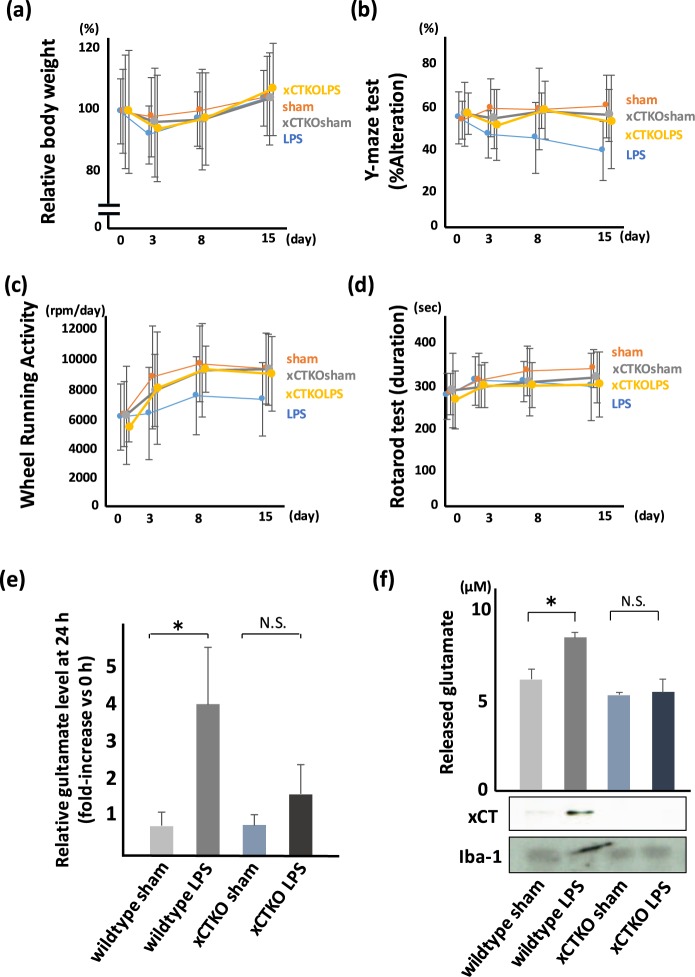
Genetical knockdown of xCT shows improved phenotype of PSNPI. Behavioral test using wildtype mice or xCT knockout mice. (**a**) BW, (**b**) Y-maze, (**c**) WRA, and (**d**) RR. (**a–d**) Wildtype sham (sham); n = 15, wildtype LPS (LPS); n = 20, xCT knockout sham (xCTKOsham; n = 14, xCT knockout LPS (xCTKOLPS); n = 14. (**a**) xCT deletion shows a tendency to improve BW loss, but the difference is not significant. F (3, 248) = 1.75, p = 0.16. (**b**) In xCT knockout mice, LPS-related memory disorder is milder than that in wildtype mice. F (3, 248) = 9.92, p < 0.0001. Post hoc; LPS vs xCTKOLPS, p < 0.005. (**c**) In xCT knockout mice, depression-like hypoactivity after LPS administration is milder than that in wildtype mice. F (3, 248) = 4.78, p < 0.005. Post hoc; LPS vs xCTKOLPS, p < 0.05. (**d**) xCT deletion shows a tendency to improve RR score, but the differences are not significant. F (3, 248) = 1.54, p = 0.20. (**e**) Comparison of glutamate level in extracellular space (*in vivo* microdialysis) at the point of 24 h after LPS treatment. Extracellular glutamate in xCT knockout mice was not significantly high (*: p < 0.01, Sham; n = 4, LPS; n = 4, xCTKOsham; n = 4, xCTKOLPS; n = 4). (**f**) Comparison of microglial glutamate release between wildtype mice and xCT knockout mice. Microglia isolated from LPS-treated xCT knockout mice do not show upregulation of glutamate (*p < 0.01, Sham; n = 5, LPS; n = 5, xCTKOsham; n = 5, xCTKOLPS; n = 5). Immunoblot for xCT and Iba-1 are also shown.

We investigated of the effect of a deficiency of xCT expression on LPS-induced behavioral disturbance using xCT knockout mice. We did not observed a significant difference in BW and RR between wildtype and xCT deficient mice treated with LPS (Fig. 4a,d), but a reduced disturbance in the test of Y-maze (Fig. 4b) and WRA (Fig. 4c) in xCT knockout mice was observed.

We examined the effect of genetic depletion of xCT on glutamate release from microglia. We measured both extracellular glutamate in the hippocampus using *in vivo* microdialysis system and released glutamate from isolated microglia. LPS-treated xCT knockout mice did not show an increase in glutamate release (Fig. 4e,f).

### Deficiency of xCT inhibits the activation of microglia and the release of inflammatory cytokines

**Figure 5:**
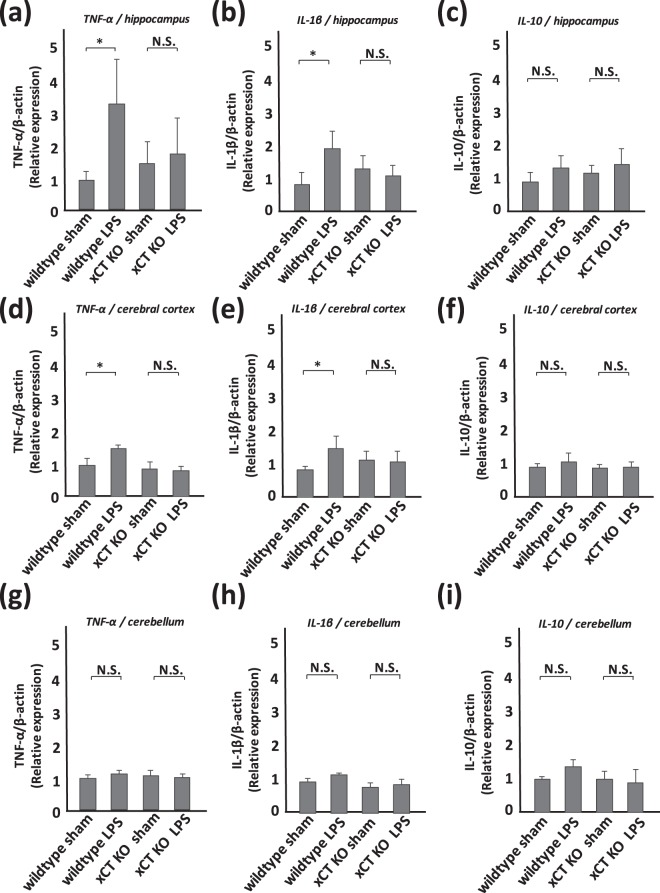
LPS-derived cytokines are inhibited in xCT knockout mice. Semi-quantitative analyses of cytokines by western blotting. (**a,d,g**) TNF-α, (**b,e,h**) IL-1β, (**c,f,i**) IL-10 in the hippocampus (**a–c**), cerebral cortex (**d–f**), and cerebellum (**g–i**). All columns indicate mean ± S.D., n = 5. In wildtype mice, TNF-α and IL-1β in the hippocampus and cerebral cortex are upregulated at 2 days after LPS treatment (*p < 0.01). The level of TNF-α and IL-1β in the cerebellum rises mildly, but the change is not significant. The level of IL-10 in each part of the brain shows a tendency for mild increase, but the differences are not statistically significant. In xCT knockout mice, each cytokine is upregulated mildly, but the change is not significant.

Proinflammatory cytokines TNF-α and IL-1β, as well as the anti-inflammatory cytokine IL-10 in the hippocampus, cerebral cortex, and cerebellum were measured in mice at 2 days after LPS treatment. Western blot analyses showed an increase of TNF-α and IL-1β in the hippocampus and cerebral cortex from LPS-treated wildtype mice (Fig. 5a,b,d,e). A significant increase of these cytokines in the hippocampus and cerebral cortex from LPS-treated xCT knockout mice was not detected (Fig. 5a,b,d,e). A tendency of an increase in TNF-α and IL-1β in the cerebellum was observed (Fig. 5g,h), but the increase was not statistically significant. A significant difference in IL-10 expression in these brain regions was not detected between sham-treated and LPS-treated groups both in wildtype and xCT knockout mice (Fig. 5c,f,i).

### The xCT inhibitor sulfasalazine improves the memory disturbance and depression-like hypoactivity induced by LPS administration

**Figure 6:**
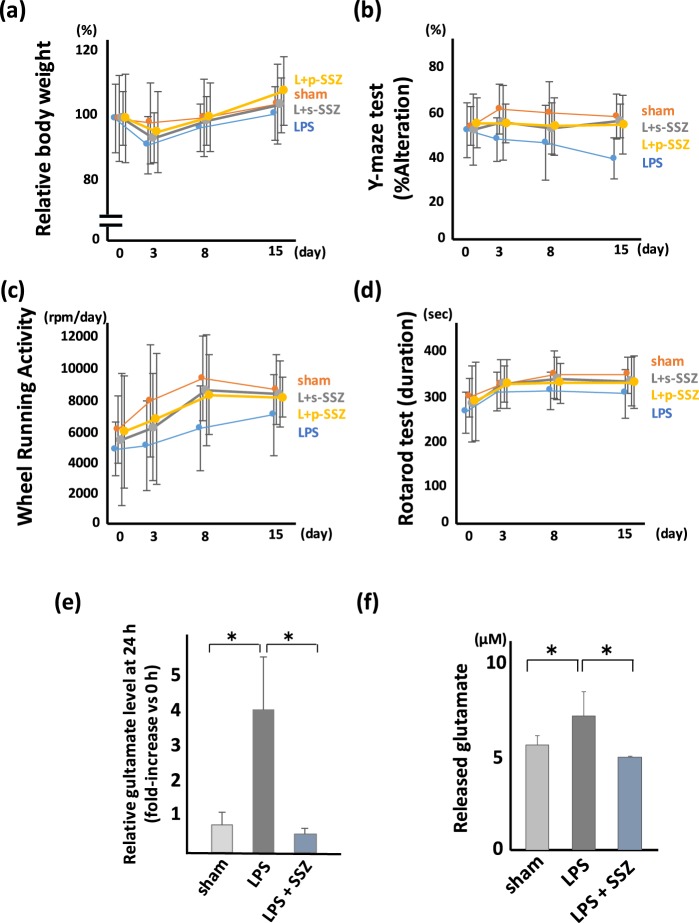
The xCT inhibitor SSZ improves the phenotype of PSNPI. Therapeutic effect of SSZ on PSNPI. (**a**) BW, (**b**) Y-maze, (**c**) WRA, and (**d**) RR. (**a**) Simultaneous treatment with SSZ and LPS (L + s-SSZ) and post-symptomatic treatment with SSZ 1 h after LPS treatment (L + p-SSZ) show a tendency to improve BW loss at 3 days after LPS treatment, but the differences are not statistically significant. F (3, 188) = 2.10, p = 0.10. (**b**) s-SSZ and p-SSZ improves the memory disorder in LPS-treated mice. F (3, 188) = 8.73, p < 0.0001. Post hoc; LPS vs L + s-SSZ, p < 0.005; LPS vs L + p-SSZ, p < 0.001. (**c**) s-SSZ and p-SSZ improves depression-like hypoactivity. F (3, 188) = 4.72, p < 0.05. Post hoc; LPS vs L + s-SSZ, p < 0.05; LPS vs L + p-SSZ, p < 0.05. (**d**) The effect of simultaneous treatment of SSZ with LPS and post-septic treatment with SSZ on the RR score is mild and is not statistically significant. F (3, 188) = 2.93, p < 0.05. Post hoc; sham vs LPS, p < 0.005; LPS vs L + s-SSZ, p = 0.051; LPS vs L + p-SSZ, p = 0.062. (**a–d**; sham; n = 11, LPS; n = 13, L + s-SSZ; n = 12, L + p-SSZ; n = 12) (**e**) Comparison of glutamate level in extracellular space (*in vivo* microdialysis) at the point of 24 h after LPS treatment. Extracellular glutamate in LPS and SSZ-treated mice was significantly lower than that in LPS-treated mice. (*p < 0.01, sham; n = 4, LPS; n = 4, L + SSZ; n = 4). (**f**) The release of glutamate from isolated microglia derived from LPS-treated mice brain is inhibited by 1 μM SSZ (*p < 0.01, sham; n = 8, LPS; n = 8, L + SSZ; n = 3).

We hypothesized that xCT may be a novel therapeutic target for memory disorder and depression-like phenotype, and investigated the effect of sulfasalazine (SSZ) on PSNPI (Fig. 6a–d). The simultaneous administration of SSZ with LPS inhibited the deterioration of recent and working memory in the Y-maze at 15 days after LPS treatment (Fig. 6b), and inhibited the hypoactivity in WRA at 8 days after LPS treatment (Fig. 6c). Moreover, the administration of SSZ at 1 h after LPS treatment, a time point when the symptoms have already appeared, also inhibited memory disturbance and hypoactivity induced by LPS administration (Fig. 6b,c). We did not observe a significant difference in BW and RR among LPS-treated mice with/without simultaneous or post-symptomatic administration of SSZ (Fig. 6a,d).

We investigated the inhibitory effect of SSZ on glutamate release by microglia from mice administered LPS. We carried out the quantification of extracellular glutamate *in vivo* and glutamate release from isolated microglia. SSZ administration significantly inhibited glutamate release from microglia (Fig. 6e,f).

We also investigated the effect of INI0602, an inhibitor of gap junction hemichannel, because it has been reported that the gap junction hemichannels are involved in glutamate release from microglia^28^. However, in our experimental conditions, neither an induction of connexin 43, one of the components of gap junction hemichannel, nor an inhibitory effect of INI0602 was detected (Supplementary Fig. S3).

## Discussion

Neurological and psychiatric illness may be observed in the delayed phase after sepsis^1,2^. These symptoms may occur even if the sepsis is not severe with no obvious necrosis of the brain tissue^7^. When the brain shows an inflammatory status including that from exposure to LPS, activated microglia are observed^12^. The importance of activation of microglia in neurological and psychiatric disorders has been received much attention in recent years, and has been reported in the brain due to infectious disease such as encephalitis, autoimmune diseases such as multiple sclerosis, and cerebrovascular diseases such as cerebral infarction or brain hemorrhage^12,29^. Similarly, the activation of microglia is observed in neurodegenerative and/or psychiatric diseases, including Parkinson’s disease, Alzheimer’s disease, amyotrophic lateral sclerosis, depression, and schizophrenia^15,16,30–32^.

Most of the studies describing microglial activation in neurological and/or psychiatric diseases report that proinflammatory cytokines or nitric oxide are critical triggers for neurological and/or psychiatric symptoms^15,16,30–32^. However, neuronal excitotoxicity induced by the excitatory amino acid glutamate is reported to be important in several neurological and psychiatric diseases^18^. Indeed, the inhibition of excitotoxicity by glutamate is used as a therapy in Alzheimer’s disease and amyotrophic lateral sclerosis^33,34^.

Using our established mouse model of memory disorder and depression-like hypoactivity after the treatment with LPS (Fig. 1), we investigated the role of glutamate in neurological and psychiatric symptoms and the sources of released glutamate. As shown in Fig. 2, the NMDA receptor antagonist MK801 and AMPA receptor antagonist DNQX reduced the symptoms induced by systemic administration of LPS. This result suggests that the recent/working memory disorder and depression-like hypoactivity are associated with extracellular glutamate. Since half-life time of these antagonists was quite short, about 2 h, MK801 or DNQX was injected not only on day 0 but also on day 1. Although it is known that NMDA antagonist can inhibit both cognitive decline and depressive symptoms, stimulation of AMPA receptor can bring an antidepressant effect^35^. Therefore, we hypothesized that AMPA antagonist DNQX could not improve the WRA score. However, DNQX improved the WRA score significantly in our experimental condition, suggesting that not only depression or anxiety but also motor function may affect this WRA score. Since microglia are activated in the brain with systemic LPS treatment, we focused on microglia as an origin of extracellular glutamate. We measured extracellular glutamate in the hippocampus using *in vivo* microdialysis technique, and we also measured glutamate released from microglia isolated from the mice with/without systemic administration of LPS. As shown in Fig. 2e,f, released glutamate from LPS-treated mice was significantly higher than that from sham-treated mice, suggesting that microglia may be an important source of extracellular glutamate in our experimental model.

Next, we focused on microglia as an origin of glutamate release in the pathogenesis of LPS-induced neurological and psychiatric disorders. Since microglia from LPS-treated mice released higher levels of glutamate than sham-treated mice, we hypothesized that an inducible mechanism for glutamate release is active in the symptomatic condition. Previous studies have identified system x_c_^−^ and gap junction hemichannel as inducible systems^27,28^. A gap junction hemichannel in microglia has been reported as an important source of glutamate release in some neurodegenerative disease. Furthermore, Takeuchi *et al*. reported that INI0602, an inhibitor of gap junction hemichannel, suppresses disease progression of Alzheimer’s disease and amyotrophic lateral sclerosis^28^. In the present study, neither inducible expression of connexin 43, one of the components of gap junction hemichannel, nor an inhibitory effect of INI0602 on glutamate release from microglia was observed (Supplementary Fig. S3). On the other hand, we detected inducible expression of xCT, a special molecule for system x_c_^−^, in microglia by both western blot analysis using microglia lysate and by immunohistochemistry (Fig. 3). Since the expression of xCT in astrocytes in LPS or 6-hydroxydopamine-treated animal model has been reported^36,37^, we also investigated the expression of xCT in astrocytes. The expression of xCT in astrocytes was also detected in later phase (Fig. 3q), but the expression level of xCT in astrocytes was lower than that in microglia. It suggests that system x_c_^−^ in microglia may play an important role in induction of PSNPI in acute or subacute phase after LPS administration but system x_c_^−^ in astrocytes may play another role especially in delayed phase.

In order to confirm the importance of system x_c_^−^ in the increase of extracellular glutamate in response to systemic administration of LPS, we examined released glutamate from isolated microglia and the behavioral analyses using xCT knockout mice. Isolated microglia derived from xCT knockout mice, even if treated with LPS, did not increase the level of glutamate release, while those derived from wildtype mice increased glutamate release. These results strongly suggest that microglial system x_c_^−^ may play a crucial role in glutamate release in the brain in response to systemic administration of LPS.

Since system x_c_^−^ is known as a transporter that induces an influx of cystine, the oxidized dimer form of amino acid cysteine, to the cells, it is very important to maintain cellular conditions under oxidative stress by supplying antioxidant GSH, which includes cysteine residue in its tripeptide^12,20^. Therefore, the absence of xCT may induce a vulnerability in microglia. To assess this point, we measured and compared the level of proinflammatory or anti-inflammatory cytokines in the brain of wildtype and xCT knockout mice. Recent studies suggest that microglia adopt two functionally distinct phenotypes, composed of phenotypes of “classical” activation (M1) and “alternative” activation (M2), and activated M1 microglia produce neurotoxic cytokines such as TNF-α and IL-1β^38,39^. Although the proinflammatory cytokines TNF-α and IL-1β were increased in the hippocampus and cerebral cortex collected from wildtype mice at 2 days after LPS administration, these increases were not observed in xCT knockout mice (Fig. 5). The expression level of the anti-inflammatory cytokine IL-10 was similar in wildtype and xCT knockout mice (Fig. 5). These expression patterns of cytokines are consistent with the neurological and psychiatric phenotype. For example, recent memory disturbance is believed to be associated with the neuronal dysfunction of the hippocampus where the increased cytokines were detected.

Our results suggest that system x_c_^−^ may be a novel therapeutic target of memory disorder and depression-like hypoactivity. SSZ is used widely in the treatment of autoimmune diseases, such as ulcerative colitis and rheumatoid arthritis^40,41^. A detailed mechanism of this compound’s effects on these diseases has not been clarified. However, a recent report described SSZ has inhibitory effect to xCT^42^. Moreover, it has been reported that SSZ can pass through the BBB via the transporter Oatp2b1^43,44^. Therefore, we suggest that SSZ administration may improve the neurological and psychiatric symptoms observed after LPS administration. Simultaneous administration of SSZ with LPS reduced memory disturbance and depression-like hypoactivity almost to the sham-treated level. Surprisingly, post-symptomatic treatment with SSZ improved those symptoms to similar levels as simultaneous treatment with SSZ (Fig. 6). These results suggest that xCT may be novel therapeutic target of neurological and psychiatric disorders in the pathogenesis of post-septic illness. Moreover, the inhibition of system x_c_^−^ may be useful for the medication of other neurological and psychiatric diseases.

## Methods

### Animals and housing

C57BL/6 mice were obtained from CLEA Japan (Tokyo, Japan). The xCT knockout mice used in this study were established and reported previously by H. Sato^45^. Since it has been reported that the morbidity of dementia and depression in women is more frequent than that in men^46^, we used female mice in this study. Wildtype (150 mice) and xCT knockout mice (50 mice) (7–8 weeks old) were used in this study. The mice were housed under standardized conditions of light (06:00–18:00), temperature (25 °C), and humidity (approximately 50%), and were allowed free access to food and water. Experiments were performed with age- and weight-matched animals. The Institutional Animal Care Committees of the Tottori University, Yonago, Japan approved all procedures (No. 16-Y-6, 18-Y-3, 19-Y-4), and all animal experiments were performed in accordance with relavant guidelines and regulations.

### Experimental design

**Figure 7:**
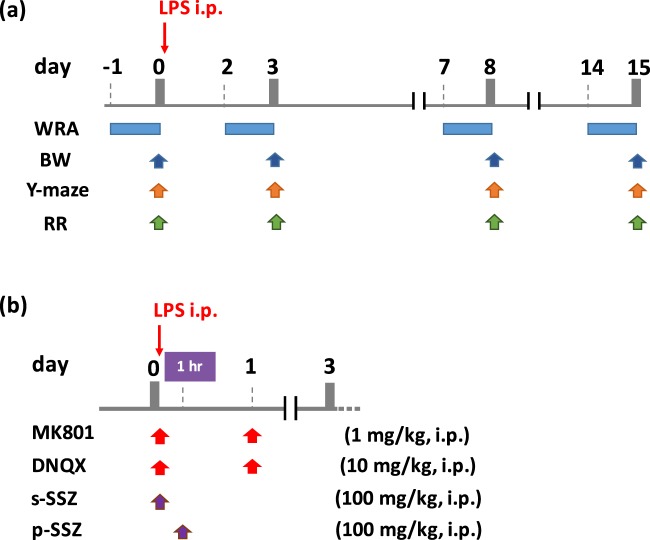
Experimental procedure. (**a**) Basic time course for LPS treatment and behavioral tests. (**b**)Timing of the administration of MK801, DNQX, and SSZ. s-SSZ means simultaneous treatment with SSZ and LPS. p-SSZ means post-symptomatic treatment with SSZ 1 h after LPS treatment.

Experimental procedures are described in Fig. 7. The mice were divided randomly into 2–4 groups, and intraperitoneal injection (i.p.) of 0.5 mg/kg BW of LPS (Sigma-Aldrich, Saint Louis, MO) was carried out [LPS group] with/without several inhibitors. For the control group, mice were administrated an equal volume of PBS (i.p.) [Sham group]. MK801, DNQX, and SSZ were obtained from Sigma-Aldrich, Tocris (Bristol, UK), and Tokyo Chemical Industry (Tokyo, Japan). The dose and timing of these chemical compounds is shown in Fig. 7.

### Behavioral test

#### Body weight (BW)

As an index of appetite, the body weight of experimental mice was measured at day 0 (before LPS administration), 3, 8, and 15 (Fig. 7a).

#### Y-maze test (Y-maze)

The Y-maze apparatus (YM-03M; Muromachi, Tokyo, Japan) was composed of three arms (10 cm wide, 13 cm high, and 35 cm long) allowing the mice to see distal spatial features^47^. The insides of the arms were identical to each other, and no interior cues were provided. Continuous spontaneous alternation testing was performed by placing the mice in the Y-maze for 8 min with all three arms. The number and sequence of arms entered were recorded manually. One alternation was counted when mice visited three different arms consecutively. Immediate re-entries were discounted. The percentage of alternation was indicated as spatial working memory. The spontaneous alternative rate (%) was calculated as the number of alternations/(the number of total arm entries − 2) × 100 (Fig. 7a).

#### Wheel running activity (WRA)

WRA was investigated using the MK-750PC (Muromachi)^47^. Locomotor and running activity was converted to revolutions per day. The mice were allowed free exercise, rest, food, and water for 24 hours (Fig. 7a).

#### Rotarod test (RR)

The motor coordination of the mice was investigated using an accelerating rotarod apparatus (MK-630B, Muromachi)^47^. The mice were placed on the rod for four successive trials. The two highest scores of the four trials were used to calculate an average score, which indicates the latency to fall. During each trial, the rotating rod accelerates gradually from 0 to 40 rpm over a period of 5 min, and, thereafter, is maintained at the 40 rpm speed (Fig. 7a).

### *In vivo* microdialysis

We measured glutamate released in extracellular space of the brain in freely moving mice using *in vivo* microdialysis system. Mice was anesthetized with mixture of medetomidine (0.3 mg/kg), midazolam (4 mg/kg), and butorphanol (5 mg/kg) (i.p.), and guide cannula (S-size: Arcrize Japan, Kurume, Japan) was implanted stereotaxically into the hippocampus (anterior: −3.8 mm, lateral: 2.0 mm, from the bregma, ventral:1.6 mm from the skull). A dialysis probe (S-size: Arcrize Japan) was inserted though the guide cannula and perfused with artificial cerebrospinal fluid (aCSF; 147 mM NaCl, 4 mM KCl, 2.3 mM CaCl_2_) at a flow rate of 1.0 μl/min. Outflow fractions (60 μl) were collected at the point of 0, 1, 3, 5, 8, and 24 h. The level of extracellular glutamate was measured by the method described below, and the results are expressed as the relative increase of glutamate levels based on the start time (0 h).

### Isolation of microglia and glutamate assay

In this study, microglia were isolated from whole brain as described previously^48^ with some modifications. Briefly, following decapitation, the whole brain except the cerebellum was readily extracted and chopped finely with a fine sharp scissor in serum-free Dulbecco’s modified Eagle’s medium (DMEM: Sigma-Aldrich) containing trypsin-EDTA (final concentrate 0.05%, Thermo Fisher Scientific, Waltham, MA), dispase II (final concentrate 2 units/ml, Roche, Basel, Switzerland), and 1% penicillin/streptomycin (Invitrogen, Carlsbad, CA). The brain pieces prepared were incubated at 37 °C for 30 min. Enzymatic digestion was terminated by adding DMEM containing 20% fetal bovine serum and 1% penicillin/streptomycin. The brain pieces were further triturated by gently pipetting and passing the tissue through a 100-μm cell strainer (Corning, Corning, NY) to remove cell debris and undigested tissue pieces. The filtered cell suspension was centrifuged at 1,000 × g for 5 min at 4 °C, and the supernatant was decanted. The cell pellet was then re-suspended by slow pipetting with 30% isotonic Percoll (GE Healthcare, Buckinghamshire, UK) in Hank’s balanced salt solution (HBSS) (Thermo Fisher Scientific) and centrifuged at 500 × g for 20 min at 4 °C. After centrifugation, the supernatant was aspirated, and the cell pellet was re-suspended in HBSS containing 0.1 μM L-cysteine (L-cysteine converts to cystine easily by autoxidation) with/without SSZ or INI0602 (Sigma-Aldrich). The eliminative process using red blood cell lysis buffer was omitted to avoid cytotoxicity.

The Glutamate-Glo^TM^ assay kit (Promega, Madison, WI) was used to measure glutamate efflux levels according to the manufacturer’s protocol. Primary microglia were suspended at 1 × 10^5^ cells/ml in HBSS containing 0.1 μM L-cysteine, then placed into 24-well culture plates. After cells were cultured at 37 °C under 5% CO_2_ in air for 30 min, the medium was collected for measurement.

### Western blotting

Brain tissues and isolated primary microglia were lysed in SDS sample buffer (50 mM Tris–HCl, pH 6.8, 2% SDS, 10% glycerol, 1 mM phenylmethylsulfonyl fluoride, and 2 mM ethylenediaminetetraacetic acid). Lysates were subjected to SDS-PAGE and then transferred onto a PVDF membranes (Hybond-P; GE Healthcare). The membrane was blocked with a 3% skim milk/PBS-Tween 0.5% (PBST) solution. The blot was then placed in the PBST solution containing anti-xCT (Abcam, Cambridge, UK), anti-Iba-1 (Abcam), anti-connexin 43 (BD Transduction Laboratories, Lexington, UK) or anti-β-actin (Cell Signaling Technology, Danvers, MA). Immunoreactive signal was detected using HRP-linked anti-rabbit or mouse IgG and ECL detection reagents (GE Healthcare) following the manufacturer’s specifications. The protein content of each sample was measured using the bicinchoninic acid (BCA) protein assay system (Thermo Fisher Scientific). The density of the immunoblot signal was semi-quantified using Image J software (National Institutes of Health, Bethesda, MD).

### Measurement of TNF-α, IL-1β, and IL-10

Semi-quantitative analyses of TNF-α, IL-1β, and IL-10 were performed by western blotting. Brain tissues (hippocampus, cerebral cortex, cerebellum) were collected at 2 or 9 days after LPS treatment. The procedure of western blotting is described above. Each antibody was purchased from Abcam.

### Histological study

HE staining and immunohistochemical analysis was performed on paraffin-embedded sections of mouse brain tissues. A portion of brain tissue was fixed by immersion in 4% paraformaldehyde (Wako, Osaka, Japan), and embedded in paraffin. Paraffin sections (5 µm) were deparaffinized by placing slides into three changes of xylene, followed by rehydration in a graded ethanol series. The sections were rinsed in water and heated by microwave in 10 mM citrate buffer (pH 6.0). The sections were incubated overnight at 4 °C in blocking reagent with the following primary antibodies: goat anti-GFAP (Abcam), goat anti-Iba-1 (Abcam), rabbit anti-xCT (Abcam). Sections were rinsed with PBS then overlaid with secondary antibodies. Peroxidase-labeled antibody was visualized using the Histo Fine kit (Nichirei, Tokyo, Japan), followed by 3,3-diamiobenzidine (DAB) reaction. Secondary immunofluorescent-labeled antibodies included mouse anti-goat IgG-FITC (Santa Cruz Biotechnology, Santa Cruz, CA) and bovine anti-rabbit IgG-TR (Santa Cruz). Nuclei were counterstained with Hoechst 33342 (ICN Biomedicals, Aurora, OH). Images were collected by fluorescent microscopy (BZ-9000; Keyence, Osaka, Japan). HE staining was performed following a standard protocol.

### Statistical analysis

Statistical analyses were performed using Statview software. Data of behavioral score were subjected two-way ANOVA, and the expression of xCT, TNF-α, IL-1β, and IL-10 were analyzed by one-way ANOVA. Bonferroni’s method was used for post hoc comparisons after ANOVA. The criterion for statistical significance was p < 0.05 for behavioral analyses, and p < 0.01 for semi-quantitative data obtained from western blotting and quantitative data obtained from glutamate assay. Absolute values are given in mean ± S.D. for behavioral analyses, semiquantitative data, and assay for released glutamate from microglia. *In vivo* microdialysis, values are given in mean ± S.E.M.

## Supplementary information

Supplementary figure
