# Supplementary material for: System xc− in microglia is a novel therapeutic target for post-septic neurological and psychiatric illness
Source: Sci Rep. 2019 May 17;9:7562. doi: 10.1038/s41598-019-44006-8 (PMC6525204; doi:10.1038/s41598-019-44006-8)
Supplement: Supplementary file 1 — Supplementary figure [file 41598_2019_44006_MOESM1_ESM.pdf]

System x_c^- in microglia is a novel therapeutic target for post-septic neurological and psychiatric illness

Yoshinori Kitagawa, Kazuhiro Nakaso, Yosuke Horikoshi, Masaki Morimoto, Takuma Omotani, Akihiro Otsuki, Yoshimi Inagaki, Hideyo Sato & Tatsuya Matsura

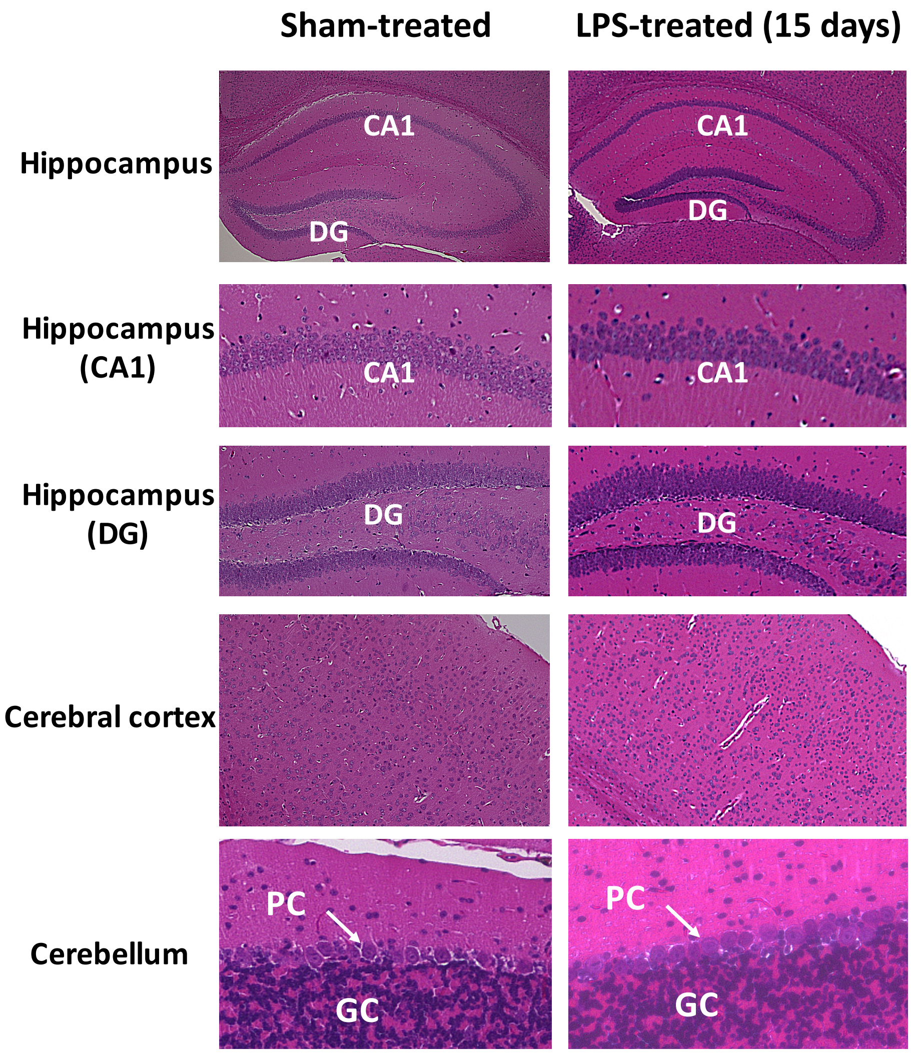

Supplementary Figure S1.
HE staining of the hippocampus, cerebral cortex, and cerebellum. Left panels show the brain section from control mice. Right panels show the section from mice treated with LPS (15 days). Obvious neuronal death is not observed. CA1: CA1 area of the hippocampus. DG: Dentate Gyrus. PC: Purkinje cell. GC: granule cells.

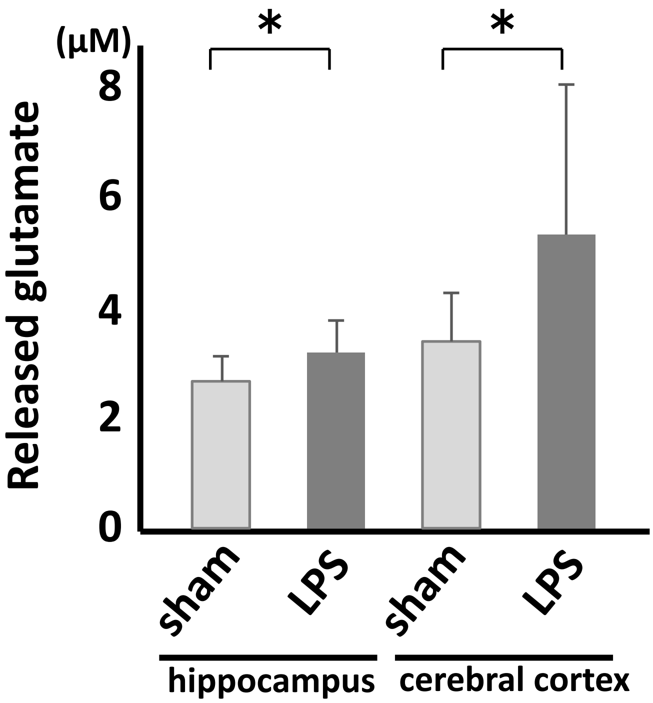

Supplementary Figure S2.

The level of glutamate released by microglia isolated from the hippocampus and cerebral corte. Released glutamate from microglia was significantly higher than that released by microglia isolated from sham-treated. *: $p < 0.05$, ANOVA.

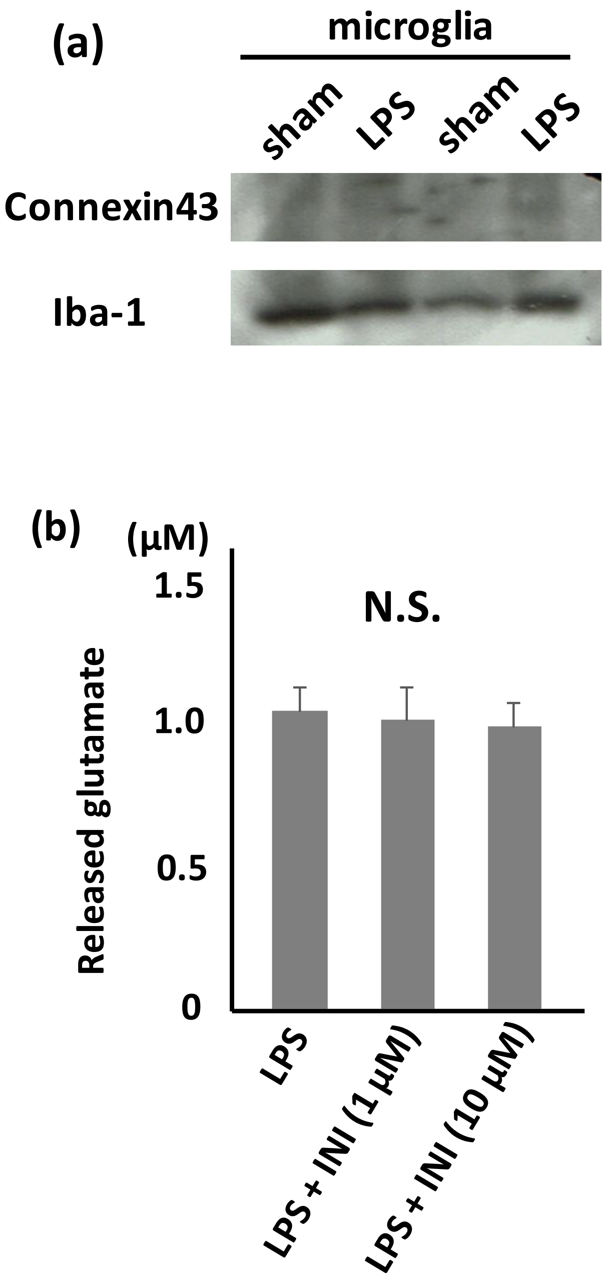

Supplementary Figure S3.

(a) Western blot analysis using connexin 43 antibody. Induction of connexin 43 in microglia derived from the brain treated with LPS is slight. (b) Comparison of microglial glutamate release among 3 groups: LPS only; LPS + INI0602 (1 μM); LPS + INI0602 (10 μM). INI0602 slightly inhibit glutamate release, but the change is not statistically significant. n = 3. 4×10^5 cells are used in each group.
